# Increased PHOSPHO1 expression mediates cortical bone mineral density in renal osteodystrophy

**DOI:** 10.1530/JOE-22-0097

**Published:** 2022-07-25

**Authors:** Shun-Neng Hsu, Louise A Stephen, Scott Dillon, Elspeth Milne, Behzad Javaheri, Andrew A Pitsillides, Amanda Novak, Jose Luis Millán, Vicky E MacRae, Katherine A Staines, Colin Farquharson

**Affiliations:** 1 The Roslin Institute and Royal (Dick) School of Veterinary Studies, University of Edinburgh, Easter Bush, Midlothian, UK; 2 Division of Nephrology, Department of Medicine, Tri-Service General Hospital, National Defense Medical Center, Taipei, Taiwan; 3 Comparative Biomedical Sciences, The Royal Veterinary College, London, UK; 4 Sanford Burnham Prebys Medical Discovery Institute, La Jolla, California, USA; 5 Centre for Stress and Age-Related Disease, University of Brighton, Brighton, UK

**Keywords:** bone mineralization, bone mineral density, chronic kidney disease-mineral bone disorder, renal osteodystrophy, PHOSPHO1, TNAP

## Abstract

Patients with advanced chronic kidney disease (CKD) often present with skeletal abnormalities, a condition known as renal osteodystrophy (ROD). While tissue non-specific alkaline phosphatase (TNAP) and PHOSPHO1 are critical for bone mineralization, their role in the etiology of ROD is unclear. To address this, ROD was induced in both WT and *Phospho1* knockout (P1KO) mice through dietary adenine supplementation. The mice presented with hyperphosphatemia, hyperparathyroidism, and elevated levels of FGF23 and bone turnover markers. In particular, we noted that in CKD mice, bone mineral density (BMD) was increased in cortical bone (*P*  < 0.05) but decreased in trabecular bone (*P*  < 0.05). These changes were accompanied by decreased TNAP (*P*  < 0.01) and increased PHOSPHO1 (*P*  < 0.001) expression in WT CKD bones. In P1KO CKD mice, the cortical BMD phenotype was rescued, suggesting that the increased cortical BMD of CKD mice was driven by increased PHOSPHO1 expression. Other structural parameters were also improved in P1KO CKD mice. We further investigated the driver of the mineralization defects, by studying the effects of FGF23, PTH, and phosphate administration on PHOSPHO1 and TNAP expression by primary murine osteoblasts. We found both PHOSPHO1 and TNAP expressions to be downregulated in response to phosphate and PTH. The *in vitro* data suggest that the TNAP reduction in CKD-MBD is driven by the hyperphosphatemia and/or hyperparathyroidism noted in these mice, while the higher PHOSPHO1 expression may be a compensatory mechanism. Increased PHOSPHO1 expression in ROD may contribute to the disordered skeletal mineralization characteristic of this progressive disorder.

## Introduction

Chronic kidney disease (CKD) is a disorder characterized by progressive loss of kidney function over time. Patients with advanced CKD frequently develop disturbances in mineral and bone metabolism and fail to maintain normal systemic levels of calcium (Ca), inorganic phosphate (Pi), parathyroid hormone (PTH), and fibroblastic growth factor-23 (FGF23) (Moe *et al.* 2006). Hyperphosphatemia, hyperparathyroidism, and elevated FGF-23 are the primary indicators for the diagnosis of CKD–mineral bone disorder (CKD–MBD) which develops in the early stages of CKD and disease progression can result in cardiovascular disease and renal osteodystrophy (ROD) – the skeletal pathology component of the CKD-MBD syndrome (Fang *et al.* 2014). The current classification system and treatment strategy for ROD are based on changes in bone turnover, mineralization, and volume (Kazama *et al.* 2013). A decrease in bone mineral density (BMD) is particularly common in patients with late-stage disease (Nickolas *et al.* 2013), but animal models have shown a more varied response (Lau *et al.* 2013, Bajwa *et al.* 2018, Metzger *et al.* 2021). The other ROD-associated skeletal pathologies have been attributed to CKD-related metabolic and hormonal disturbances (Zheng *et al.* 2016).

Although the precise mechanisms responsible for the impaired skeletal mineralization observed in ROD remain unclear, the origins are likely to involve a complex interplay between bone and the altered endocrine milieu. Phosphorus retention, due to the failing kidney, leads to chronically elevated concentrations of circulating FGF23 in an attempt to normalize serum Pi levels through enhanced urinary secretion and decreased intestinal absorption (Mirza *et al.* 2009). This is achieved by the inhibition of renal 1α-hydroxylase and stimulation of 24-hydroxylase, but the resulting reduction in circulating levels of 1,25(OH)_2_D_3_ contributes to hypocalcemia and secondary hyperparathyroidism (SHPT) (Shimada *et al.* 2004). SHPT promotes bone resorption by increasing the receptor activator of nuclear factor-ƙB ligand (RANKL)/osteoprotegerin (OPG) ratio (Ma *et al.* 2001). The bone formed during rapid remodeling is both immature and poorly mineralized (Graciolli *et al.* 2017). Indeed, the mineralization status may be dependent on the prevailing serum PTH concentrations which could explain the various mineralization states reported in ROD (Miller *et al.* 1998, Lau *et al.* 2013). It is also possible that altered endocrine factors may directly target the expression of key phosphatases critical for skeletal mineralization. Specifically, FGF23 may inhibit matrix mineralization by suppressing TNAP expression and activity by osteoblasts resulting in the accumulation of the mineralization inhibitor, pyrophosphate (PPi) (Murali *et al.* 2016*b*). Also, PTH may induce a rapid downregulation of *Phospho1* gene expression in osteogenic cells and bone marrow stromal cell lines (Houston *et al.* 2016, Chande & Bergwitz 2018). Despite clear links between both TNAP and PHOSPHO1 in the control of skeletal mineralization, their roles in ROD remain unclear.

PHOSPHO1 and TNAP are two of the most widely studied phosphatases involved in skeletal mineralization (Dillon *et al.* 2019). PHOSPHO1 is expressed at the sites of mineralization and liberates Pi from phospholipid substrates for incorporation into the mineral phase (Roberts *et al.* 2007).* Phospho1-*deficient mice exhibit decreased BMD, compromised trabecular and cortical bone microarchitecture, and spontaneous greenstick fractures (Boyde *et al.* 2017). TNAP is an ectoenzyme and hydrolyzes PPi to allow the propagation of hydroxyapatite in the extracellular matrix (ECM), beyond the confines of the matrix vesicle membrane (Hessle *et al.* 2002). Mice deficient in TNAP (*Alpl^−/−^*) phenocopy infantile hypophosphatasia (HPP), an inborn error of metabolism resulting in rickets and osteomalacia (Whyte 2008). A complete absence of ECM mineralization is observed in *Phospho1^−/−^; Alpl^−/−^* double knockout mice and in murine metatarsals cultured in the presence of PHOSPHO1 and TNAP inhibitors demonstrating the functional co-operativity of PHOSPHO1 and TNAP for bone mineralization (Yadav *et al.* 2011, Huesa *et al.* 2015).

Despite great advances in understanding the mechanisms responsible for the altered mineralization status noted in ROD, the involvement of phosphatases is unclear. Therefore, in this study, we examined changes in the expression of PHOSPHO1 and TNAP and bone architecture in long bones using the well-established adenine-induced model of CKD (Jia *et al.* 2013). We also examined the effects of PTH, FGF23, and Pi on TNAP and PHOSPHO1 expression in primary osteoblasts. Our findings support a specific role for PHOSPHO1, particualarly, in the altered cortical bone mineralization status in ROD.

## Materials and methods

All reagents were from Sigma–Aldrich (Gillingham, Dorset, UK) or less otherwise stated.

### Mice

C57BL/6 male mice (Charles River Laboratories) were used in the first *in vivo* study. Male *Phospho1* knockout (P1KO) mice and WT controls, maintained on a C57BL/6 background were generated as previously described (Yadav *et al.* 2011) and used in the second *in vivo* study. At 8 weeks of age, mice were randomly assigned a control (*n*  = 12) or CKD (*n*  = 12) diet (Supplementary Fig. 1A, see section on supplementary materials given at the end of this article). Mice losing more than 30% of their body weight were sacrificed by exposure to CO_2_ and confirmed dead by cervical dislocation. All animal experiments were approved by the Roslin Institute’s named veterinary surgeon and named animal care and welfare officer (NACWO), with animals maintained in accordance with the Home Office code of practice (for the housing and care of animals bred, supplied, or used for scientific purposes).

### CKD diet and tissue collection

CKD was induced by feeding a casein-based diet containing 0.6% calcium, 0.9% phosphate, 1.5% Vitamin Mix, AIN-76A (containing vitamin D_3_), and 0.2% adenine (catalog number: TD.140290, Envigo, Teklad Co. Ltd). Control mice received the same diet without adenine (catalog number: TD.138898, Envigo). All mice were fed their respective diets for 5 weeks and at 13 weeks of age, all animals were sacrificed, and blood was obtained by cardiac puncture under terminal anesthesia. Femora, tibiae, and kidneys were harvested, processed, and stored accordingly.

### Serum and urine biochemistry

Serum blood urea nitrogen (BUN), creatinine (Cr), Ca, Pi, and alkaline phosphatase (ALP) activity were quantified using a biochemistry analyzer (Beckman Coulter AU480). Intact PTH (Pathway Diagnostics, Dorking, UK), FGF23 (Kainos Laboratories, Inc. Japan), N-terminal propeptide of human procollagen type I (P1NP), and carboxy-terminal telopeptide of type I collagen (αCTX) (Wuhan Fine Biotech, Wuhan, China) levels were determined by ELISA according to manufacturers’ instructions. Hydrophobic bedding, LabSand (Coastline Global, CA, USA), was used to collect urine samples from which the concentration of Cr and albumin were determined by semi-quantitative test strips (Microalbustix, Siemens) and the specific gravity (SG) was determined by a manual refractometer.

### Histopathological analysis of kidney and bone tissues

The right tibiae and kidneys were fixed in 4% paraformaldehyde (PFA, for 24 h) and stored in 70% ethanol. Kidneys were processed in paraffin wax using standard procedures. Hematoxylin and eosin, Masson’s trichrome, and von Kossa staining were performed according to standard methods. Histopathological scoring of renal interstitial inflammation, tubular atrophy, protein casts, and renal fibrosis was defined as 0 = normal; 1 = mild, involvement of <25% of the cortex; 2 = moderate, involvement of 25–50% of the cortex; 3 = severe, involvement of 50–75% of the cortex; 4 = extensive, involvement of >75% of the cortex. Bones were decalcified in 10% ethylenediaminetetraacetic acid (EDTA; pH 7.4) for 14 days at 4°C and processed in paraffin wax. Sections were stained using Goldner’s Trichrome and reacted for tartrate-resistant acid phosphatase. Bone histomorphometry was quantified using the BioQuant Osteo software (BIOQUANT Image Analysis Corporation, Texas, USA) using the approved ASBMR histomorphometry nomenclature (three sections/bone: six randomly selected bones from each group).

### Micro computed tomography

The bone structure of the left tibiae was determined using micro-CT (μCT, Skyscan 1172, Bruker, Kontich, Belgium). High-resolution scans with an isotropic voxel size of 5 μm were acquired (60 kV, 167 μA, and 0.5 mm filter, 0.6° rotation angle) and from the reconstructed images (NRecon 1.7.3.0 program; Bruker), CTAn software 1.15.4.0 (Skyscan) was used to visualize and determine bone histomorphometric parameters. Three-dimensional images were created using IMARIS 9.0.

In the proximal tibial metaphysis, the volume of interest extended distally 5% from the bottom of the growth plate excluding the cortical shell. A total of 250 slices beneath this 5% were selected to exclude the primary spongiosa. In the first *in vivo* study, whole bone cortical analysis was performed on data sets derived from whole μCT scans using BoneJ (version 1.13.14), a plugin for ImageJ. Following segmentation, alignment, and removal of fibula from the data set, a minimum bone threshold was selected for each bone to separate higher density bone from soft tissues and air. The most proximal and the most distal 10% portions of tibial length were excluded from analysis, as these regions include trabecular bone. In the second *in vivo* study, cortical analysis was performed on data sets derived from μCT scan images at 50% of the total tibial length from the top of the tibia. BMD phantoms of known calcium hydroxyapatite mineral densities of 0.25 and 0.75 g/cm^3^ were scanned and reconstructed using the same parameters as used for bone samples.

### Primary calvarial osteoblast isolation and culture

Calvarial osteoblasts were obtained from 3- to 5-day-old C57BL/6 mice by sequential enzyme digestion (1 mg/mL collagenase type II (Worthington Biochemical, Lakewood, NJ, USA) in Hanks’ balanced salt solution (Life Technologies); 4 mM EDTA). The cells were grown in α-minimum essential medium (αMEM, Invitrogen) supplemented with 10% fetal bovine serum and 0.5% gentamycin (Life Technologies) until confluent.

### Establishment of Pi substrate-free mineralization model for primary osteoblast culture

To study the effects of varying Pi concentrations on phosphatase expression, it was essential to control Pi concentration in the basal mineralizing medium. This ruled out the use of β-glycerophosphate (βGP) as the availability of Pi from βGP requires the action of TNAP (Huesa *et al.* 2015) which can itself be modulated by CKD-associated endocrine factors such as Pi, PTH, and FGF23 (Shalhoub *et al.* 2011, Rendenbach *et al.* 2014, Houston *et al.* 2016). Therefore, upon confluence (day 0), mineralization was induced by supplementing the growth medium (basal concentration: 1.8 mM Ca; 1 mM Pi) with 50 μg/mL l-ascorbic acid (AA) and 1.5 mM CaCl_2_ to provide a final medium containing 3.3 mM Ca (Houston *et al.* 2016). Cultures were also supplemented with a range of Pi (1–5 mM), PTH (0–50 nM), and FGF23 (0–200 ng/mL) with or without klotho (50 ng/mL) (R&D Systems). Cells were maintained in a 5% CO_2_ atmosphere at 37°C and mineralization media was changed every second/third day for 28 days.

### Cell viability and cytotoxicity assay

To assess the effects of Pi on osteoblast viability, the AlamarBlue assay (Thermo Fisher Scientific) and lactate dehydrogenase (LDH) CytoTox 96 cytotoxicity assay (Promega) were performed according to manufacturer’s instructions.

### RNA extraction and quantitative polymerase chain reaction

The distal and proximal epiphyses of the left femoral were excised, and the diaphyseal bone marrow was removed by centrifugation at 13,000 ***g*** for 10 min at 4°C. The resultant cortical shafts were homogenized using a Rotor-Stator Homogenizer (Ultra-Turrax T10). RNA extraction from the homogenized bone and cultured osteoblasts was completed using the RNeasy kit (Qiagen). The RNA concentration was determined using a NanoDrop spectrophotometer (Fisher Scientific) at a wavelength of 260 nm, and RNA purity was evaluated by the 260/280 nm ratio. RNA was reverse transcribed to cDNA using Superscript II (Invitrogen). All genes were analyzed with the SYBR green detection method (PCR Biosystems, UK) using the Stratagene Mx3000P real‐time QPCR system (Agilent Technologies). Gene expression data were normalized against housekeeping genes (*Gapdh* in primary osteoblasts and* Atp5b* in bone tissue) using MxPro software (Agilent Technologies). The relative expression of the analyzed genes was calculated and expressed as a fold change compared to control values. Primer sequences are listed in Supplementary Table 1.

### Protein extraction and isolation from brush border membrane vesicles of kidney

Kidneys were homogenized in ice-cold buffer A (50 mM D-mannitol, 2 mM HEPES, 2.5 mM ethylene glycol-bis (2-aminoethylether)-N, N, N', N'-tetraacetic acid (EGTA), and 12 mM Tris-base titrated to pH 7.1) and mixed with a protease inhibitor cocktail. Brush border membrane vesicles (BBMVs) were isolated from microvilli of kidneys using two consecutive magnesium precipitations in buffer A and then buffer B (150 mM D-mannitol, 2.5 mM EGTA, and 6 mM Tris hydrochloride). The resultant BBMV pellet was resuspended in radioimmunoprecipitation assay (RIPA) buffer (Thermo Fisher Scientific) containing a protease inhibitor cocktail.

### Western blot analysis

Protein from cultured osteoblasts and right femoral diaphyseal cortical bone (with marrow removed) was extracted in RIPA buffer containing protease inhibitor cocktail after homogenization. Protein concentrations were determined using the bicinchoninic acid (BCA) protein assay kit (Life Technologies). Proteins were separated using a 10% Bis-Tris protein gel (Thermo Fisher Scientific). After blocking in 5% skimmed milk/Tris-buffered saline with Tween 20 or LI-COR buffer at room temperature (RT) for 1 h, the membranes were incubated sequentially with primary and secondary antibodies (Supplementary Tables 2 and 3). Western blot analysis of proteins from primary osteoblasts was performed using the Odyssey infrared detection system (LI-COR). Western blot analysis of proteins from bone tissues was undertaken using the ultra-sensitive ECL detection system (Thermo Fisher Scientific). The blots were imaged by the GeneGnome XRQ chemiluminescence imaging system (Syngene, Cambridge, UK). Densitometry of the protein bands was analyzed with ImageJ software (NIH) for quantification.

### Quantification of ECM mineralization

Cultured osteoblasts were fixed in 4% PFA for 10 min at RT and stained with aqueous 2 % (w/v) Alizarin red solution for 10 min at RT. The bound stain was solubilized in 10% cetylpyridinium chloride and the optical density was measured by spectrophotometry at 570 nm.

### Statistical analysis

Quantitative data are expressed as the mean ±s.e.m. of at least three biological replicates per experiment. The precise number (*n*) is indicated in the relevant table and figure legends. Statistical analysis was performed using a two-tailed Student’s *t*-test or one-way ANOVA followed by Tukey’s range test, as appropriate. Statistical analysis was implemented by the GraphPad Prism software. A *P* < 0.05 was considered to be significant and noted as ‘*’. *P* values of <0.01, <0.001, and <0.0001 were noted as ‘**’, ‘***’, and ‘****’, respectively.

## Results

### Verification of the CKD mouse model

Before investigating TNAP and PHOSPHO1 expression in experimental ROD, we first confirmed that our mouse model presents with the characteristic serum biochemistries and kidney pathologies of CKD. The CKD mice lost bodyweight and presented with the expected changes to serum and urine analyte levels at the end of the study (Table 1 and Supplementary Fig. 1B). The kidneys of CKD mice presented with various pathologies including tubular atrophy, protein casts, interstitial inflammation, and renal fibrosis (Supplementary Fig. 2). Furthermore, transcripts encoding kidney injury biomarkers *Lcn2* (protein; Ngal) and *Spp1* (protein; osteopontin (OPN)) (Viau *et al.* 2010, Kaleta 2019), as well as *Fgf23*, were increased in CKD mice, whereas *Slc34a1* (protein; NaPi-2a) expression was decreased (Supplementary Fig. 3A). Protein expression of OPN and NaPi-2a by BBMV confirmed the transcript data (Supplementary Fig. 3B). Collectively, these data confirm previous reports that mice fed an adenine-rich diet for 5 weeks developed CKD (Tamura *et al.* 2009, Jia *et al.* 2013, Metzger *et al.* 2020).

**Table 1:** Body weight, serum, and urine biochemistries in control and CKD mice.

Parameters	CONTROL (*n* = 12)	CKD (*n* = 8)	*P* value
Body weight (g)	27.07 ± 1.81	19.06 ± 2.75	<0.0001
Serum			
BUN (mg/dL)	25.51 ± 1.18	65.16 ± 1.82	<0.0001
Cr (mg/dL)	0.33 ± 0.00	0.58 ± 0.01	<0.0001
Ca (mg/dL)	9.70 ± 0.15	10.65 ± 0.18	<0.0001
Pi (mg/dL)	9.02 ± 0.24	13.32 ± 0.62	<0.0001
ALP (IU/L)	162.92 ± 22.06	120.80 ± 6.01	NS
PTH (pg/mL)	1070.35 ± 154.40	1967.13 ± 204.40	<0.01
FGF23 (ng/mL)	0.32 ± 0.05	47.74 ± 4.56	<0.0001
Total P1NP (ng/mL)	0.42 ± 0.08	0.78 ± 0.09	<0.01
αCTx (ng/mL)	6.19 ± 1.86	16.45 ± 1.97	<0.01
Urine			
Cr (mg/dL)	262.50 ± 18.30	62.50 ± 8.18	<0.0001
Albumin (mg/L)	141.25 ± 8.75	42.50 ± 8.18	<0.0001
SG	>1.04 ± 0.00	1.02 ± 0.00	<0.0001

Four mice on the CKD diet lost > 30% bodyweight between 4 and 5 weeks and were removed from the study. The data are represented as the means ± s.e.m.

ALP, alkaline phosphatase; BUN, blood urea nitrogen; Ca, calcium; Cr, creatinine; FGF23, fibroblast growth factor 23; Pi, phosphorus; PTH, parathyroid hormone; SG, specific gravity.

### PHOSPHO1 and TNAP expressions are altered in the bones of CKD mice

**Figure 1:**
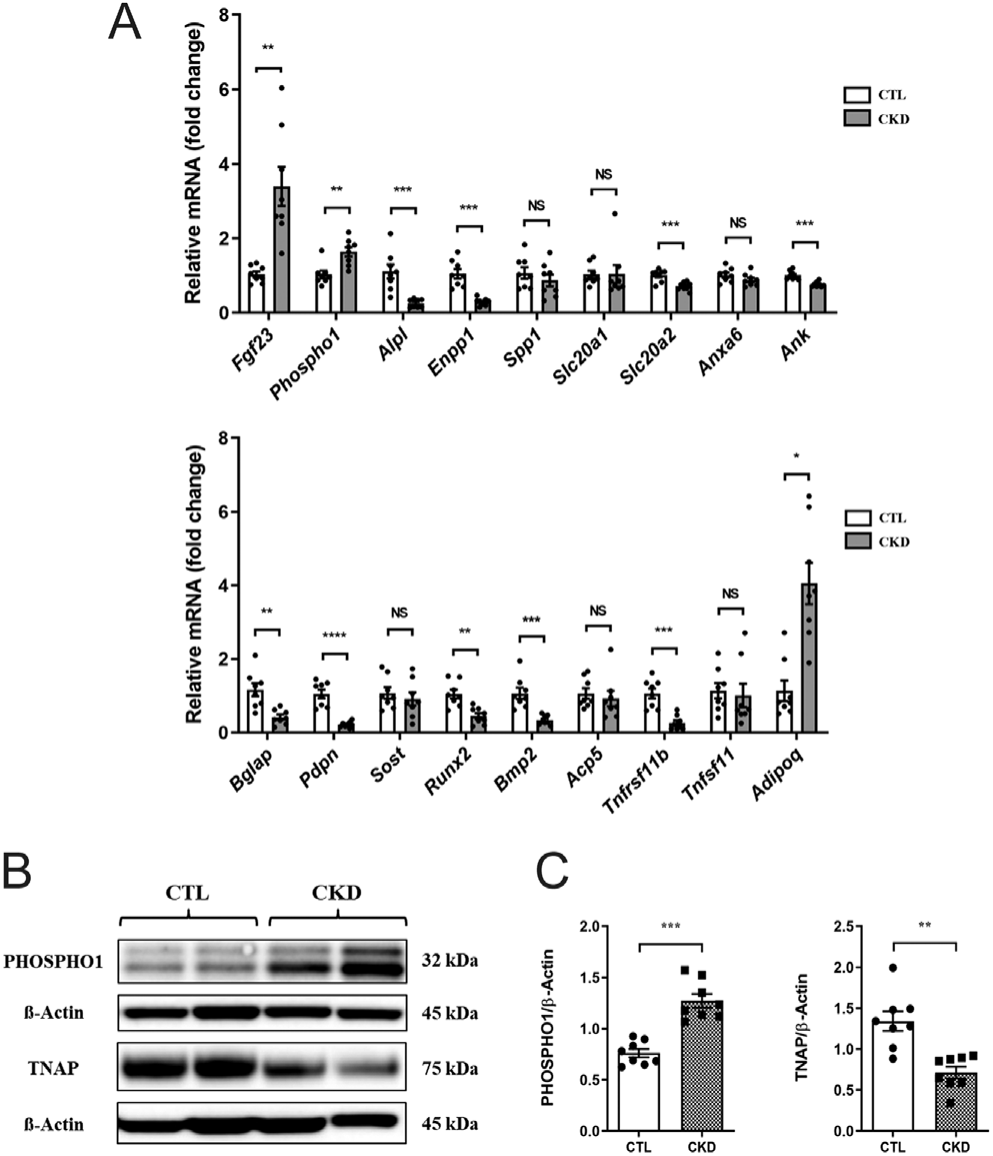
Expression of osteoblast and mineralization markers in mouse femurs from CTL and CKD mice. (A) *E*xpression of key mineralization and osteoblast marker genes in femurs of CTL and CKD mice at the end of the study (13 weeks of age). Of note, *Fgf23* and *Phospho1* expressions were increased and *Alpl* expression was decreased in the femurs of the CKD-MBD mice. (B) Representative image of 2 CTL and 2 CKD-MBD femurs analyzed by Western blot for PHOSPHO1 and TNAP expression. (C) Quantification of PHOSPHO1 and TNAP expression indicated that PHOSPHO1 was increased and TNAP was decreased in the femur of CKD-MBD mice compared with control mice. The data are represented as the mean ± s.e.m. (*n*  = 8); ^*^P < 0.05; ^**^P < 0.01;^ ***^P < 0.001; ^****^P < 0.0001.

*Phospho1* expression was increased and *Alpl* expression was decreased in the femur of CKD mice when compared to control mice. The expression of *Enpp1*, *Slc20a2*, *Ank*, *Bglap*, *Pdpn*, *Runx2*, *Bmp2*, *Npnt*, *and Tnfrsf11b* was decreased, whereas femoral expression of *Fgf23,* and *Adipoq, was* increased in CKD mice when compared to control mice (Fig. 1A). The changes in *Phospho1* and *Alpl* expression in femurs of CKD mice were confirmed at the protein level (Fig. 1B and C).

### Cortical BMD is increased in CKD mice and is influenced by PHOSPHO1 status

**Figure 2:**
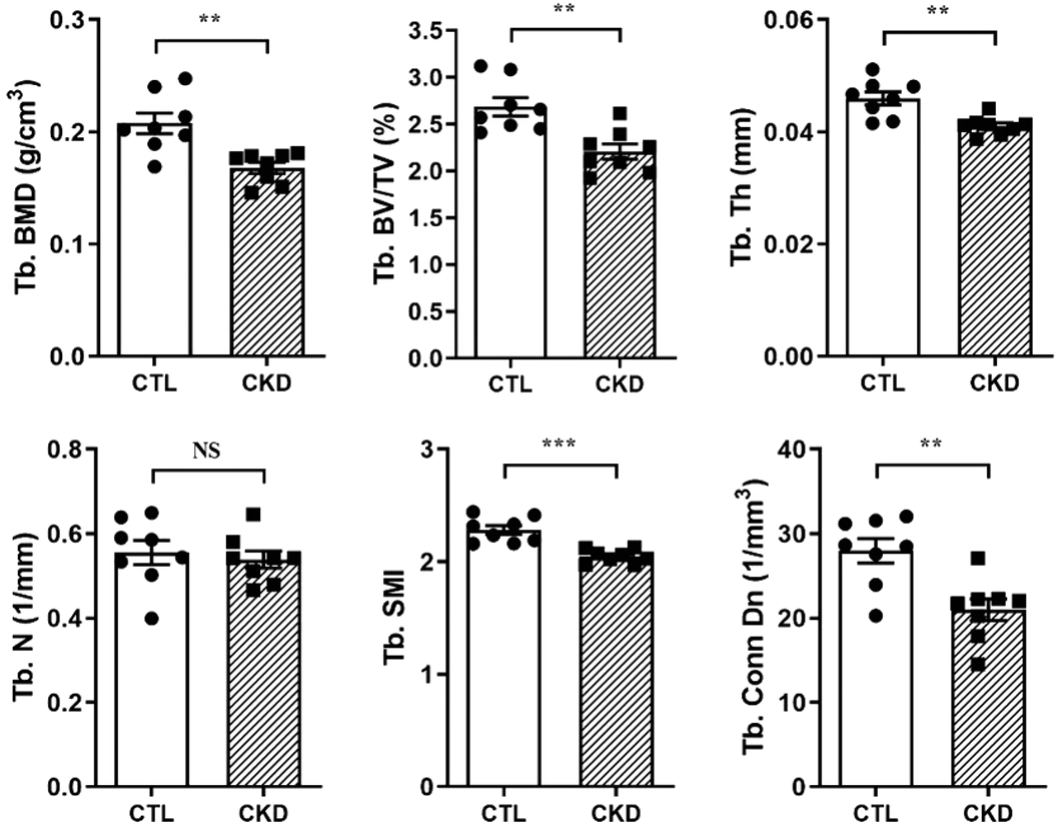
Micro-CT analysis of trabecular bone of the tibia. Micro-CT analysis of tibia from male C57BL/6 mice fed a CTL or CKD diet for 5 weeks. Tb. BMD (trabecular bone mineral density; g/cm^3^); Tb. BV/TV (trabecular bone volume/tissue volume; %); Tb. Th. (trabecular thickness; mm); SMI (structure model index); Tb. Conn Dn (trabecular connectivity density; mm^−3^) were all decreased in the CKD-MBD mice. Tb. N. (trabecular number; mm^−1^) was unchanged. Tibia of *n*  = 8 (CTL mice) vs *n*  = 8 (CKD-MBD mice) biological replicates was analyzed. The data are represented as the means ± s.e.m. ^*^P < 0.05; ^**^P < 0.01; ^***^P < 0.001 vs CTL.

**Figure 3:**
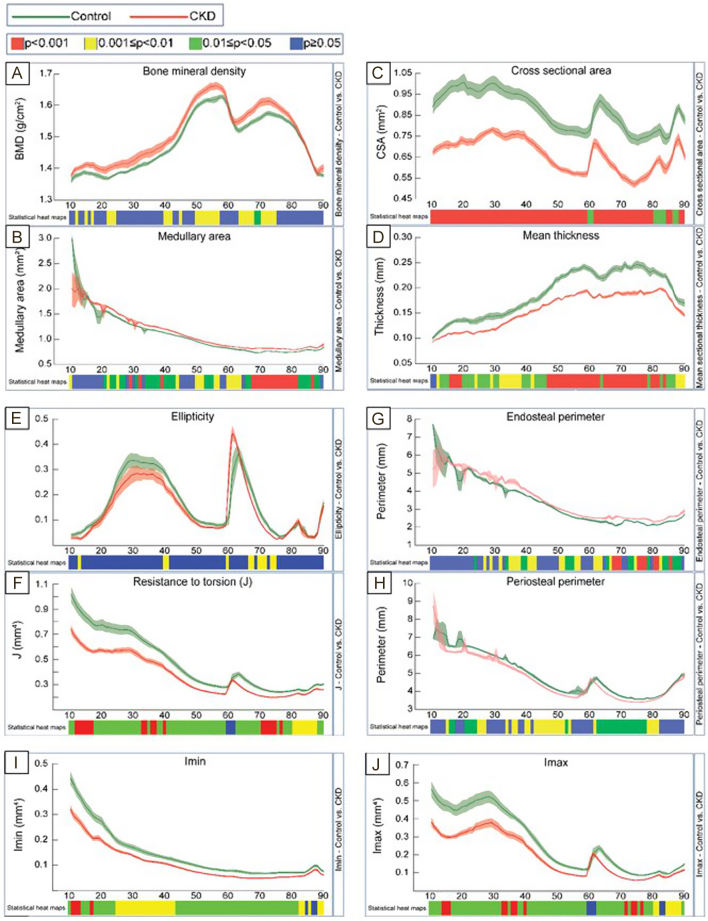
Micro-CT analysis of whole cortical bone of the tibia. Micro-CT analysis of tibia from male C57BL/6 mice fed a CTL or CKD diet for 5 weeks. Quantification of whole bone analyses of cortical bone between 10 and 90% of total tibial length, excluding proximal and distal metaphyseal bone, of CTL and CKD tibia at 13 weeks of age. (A) BMD (bone mineral density; g/cm^3^), (B) medullary area (cm^2^), and (G) endosteal perimeter (mm) were generally increased and (C) CSA (cross-sectional area; mm^2^), (D) mean thickness (mm), (F) resistance to torsion (J; mm^4^), (H) periosteal perimeter (mm), (I) Imin (mm^4^), and (J) Imax (mm^4^) were generally decreased in the CKD–MBD bones. Tibia of *n*  = 8 (CTL mice) vs *n*  = 8 (CKD mice) biological replicates was analysed. *P* < 0.05 was significant and *P* ≤ 0.01–0.05 was give in green, *P* ≤ 0.001–0.01 in yellow, and* P* ≤ 0.000–0.001 in red. Not significant is given in blue.

Trabecular BMD, bone volume/tissue volume (BV/TV), thickness (Th), structural model index, and connectivity density of the tibiae were all decreased in CKD mice when compared to controls (Fig. 2). Cortical bone parameters were also altered in CKD mice; cortical BMD was increased in discrete regions, whereas cross-sectional area (CSA), cortical thickness, resistance to torsion, and Imin and Imax were all generally lower over the entire tibial length of CKD mice (Fig. 3A, C, D, F, I and J). Consistent with the thinner cortex, the medullary area and the endosteal perimeter were increased and the periosteal perimeter decreased in the CKD mice (Fig. 3B, D, G and H). The histomorphometric analysis is consistent with the reduced trabecular BV/TV in the CKD mice (Supplementary Fig. 4A, i, ii and B). The osteoid volume/bone volume (OV/BV) was increased in CKD mice confirming the impaired mineralization in this compartment (Supplementary Fig. 4A, iii, iv and B). Osteoclast number associated with trabecular bone within the primary spongiosa of CKD mice was increased (Supplementary Fig. 4A, v, vi and B); an observation consistent with decreased *Tnfrsf11b* (osteoprotegerin) expression in CKD bones (Fig. 1A) and higher serum αCTX concentrations in CKD mice (Table 1).

**Figure 4:**
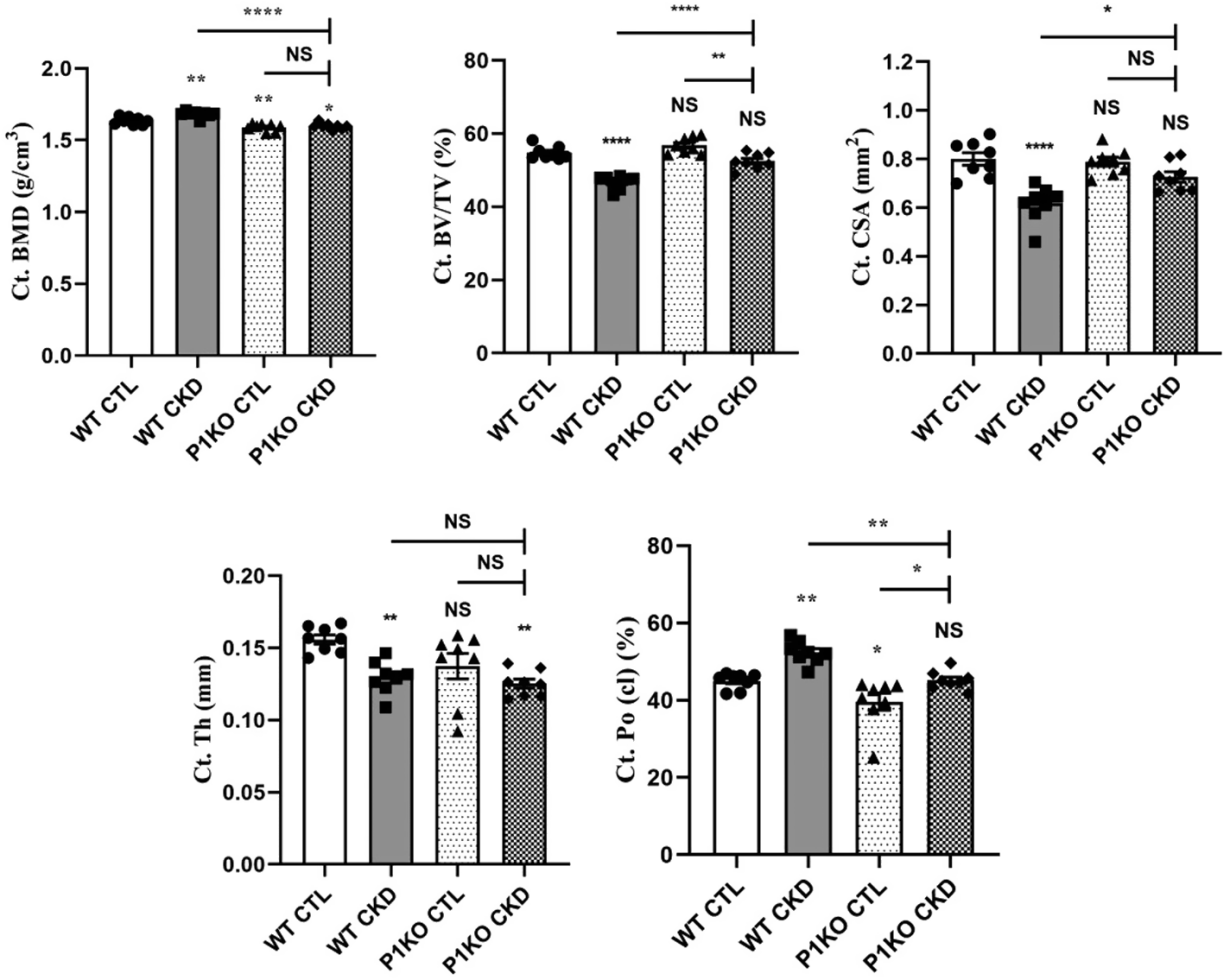
Micro-CT analysis of cortical bone of WT and PHOSPHO1-deficient CTL and CKD mice. Quantification of cortical bone mineral density (Ct. BMD), cortical bone volume/tissue volume (Ct. BV/TV), cortical cross-sectional area (Ct. CSA), cortical thickness (Ct. Th), and closed pore porosity (Ct Po (cl)) at 50% of the total tibial length from the top of the tibia. Of note, BMD was increased in the WT CKD-MBD tibia but not in the PHOSPHO1-deficient CKD–MBD tibia when compared to their respective controls. The data are represented as the mean ± s.e.m. (*n*  = 8); ^*^P < 0.05; ^**^P < 0.001; ^****^P < 0.0001 compared to WT CTL bones.

The increased cortical BMD in CKD–MBD mice (Fig. 3A) aligns with the higher PHOSPHO1 expression in the cortical bone shafts, despite being an unexpected finding in the CKD–MBD mice (Fig. 1A, B and C). To explore this further, we next examined bone from PHOSPHO1-deficient (P1KO) mice maintained on 0.2% adenine supplemented diet for 5 weeks. Cortical analysis was performed on data sets derived from μCT scan images at 50% of the total tibial length as this region of bone from CKD mice had a higher BMD than control counterparts (Fig. 3A). As previously noted (Fig. 3A), the cortical BMD of WT CKD mice was increased compared to WT control mice, but, in contrast, no such increase was apparent in P1KO CKD mice, which had a BMD similar to their respective P1KO controls but as expected, lower than the BMD of WT control mice (Fig. 4). Structural cortical bone changes were also influenced by the absence of PHOSPHO1 in the P1KO mice; the CKD-induced increases in porosity and decreases in BV/TV, CSA, and Th noted in WT CKD mice were all blunted in P1KO CKD mice compared to P1KO control mice (Fig. 4). The response of trabecular bone in mice with CKD was similarly affected by PHOSPHO1 status (Supplementary Fig. 5).

### Pi, PTH, and FGF23 perturb ECM mineralization and the expression of key mineralization markers in primary osteoblasts

To investigate the causes of the mineralization defects noted in the CKD mice, we investigated the direct effects of FGF23, PTH, and Pi on the expression of PHOSPHO1 and TNAP and other key regulators of mineralization by primary osteoblasts in cultures. Over 28 days, the basal Pi substrate-free mineralization media promoted matrix mineralization (Supplementary Fig. 6A and B) and PHOSPHO1 and TNAP expressions in a temporal manner at both the gene and protein level confirming the suitability of this culture model for our purposes (Supplementary Fig. 6C, D and E).

**Figure 5:**
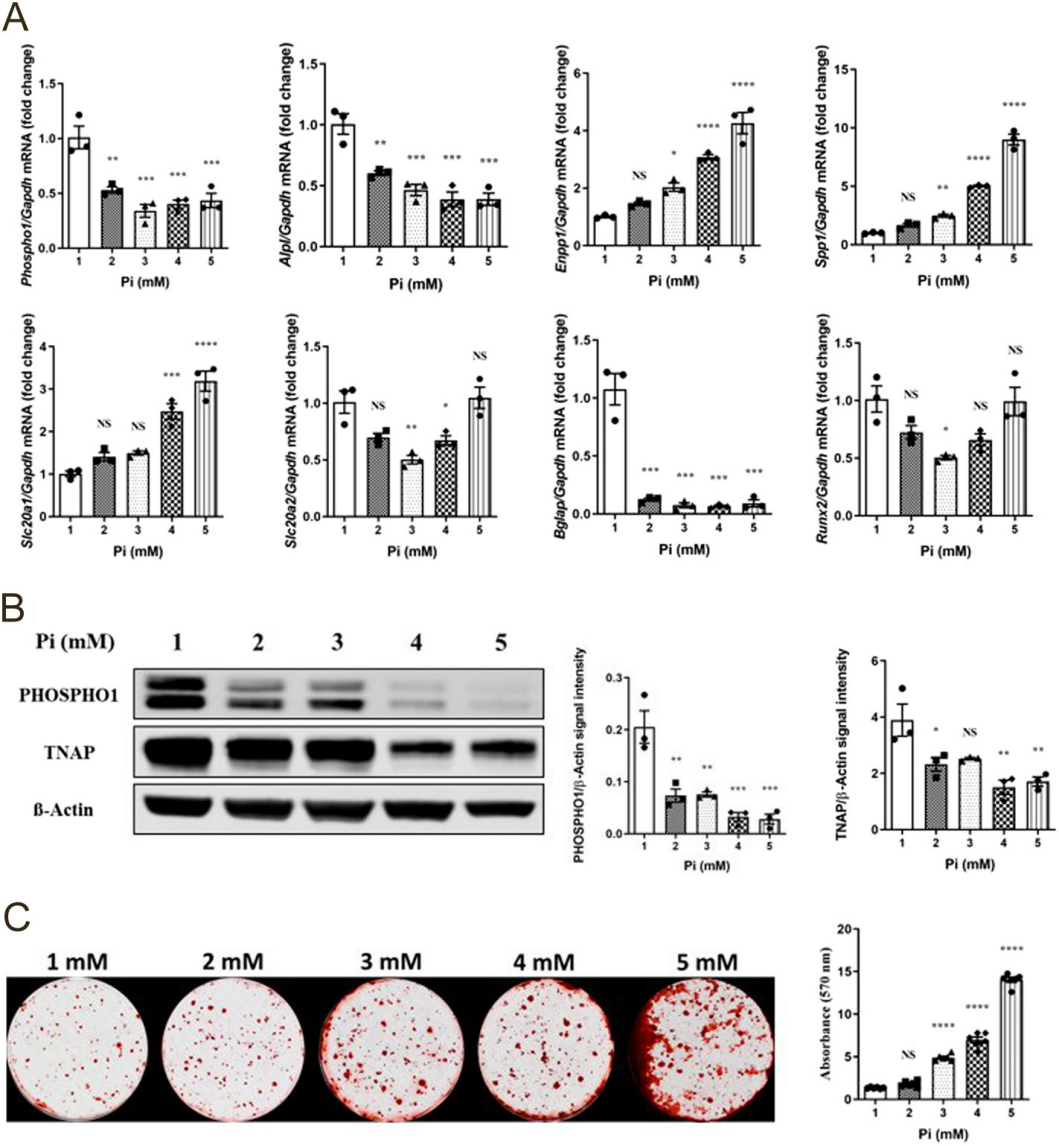
Regulation of key mineralization associated genes, proteins, and osteoblast extracellular matrix mineralization by Pi in primary osteoblasts. (A) Expression analysis of *Phospho1*, *Alpl*, *Enpp1*, *Spp1, Slc20a1, Slc20a2, Bglap, and Runx2* by osteoblasts in response to Pi (1–5 mM), (B) Western blotting analysis and quantification of PHOSPHO1 and TNAP expression in response to Pi, and (C) representative images and quantification of alizarin red staining in response to Pi for 28 days after confluency. PHOSPHO1 and TNAP at the gene and protein level were decreased with increasing Pi concentrations, whereas matrix mineralization increased with increasing Pi concentrations. The data are represented as the mean ± s.e.m. (*n*  = 3); ^*^P < 0.05; ^**^P < 0.01; ^***^P < 0.001; ^****^P < 0.0001 compared to 1 mM Pi cultures.

At concentrations of 2 mM and above, Pi significantly downregulated *Phospho1, Alpl, and Bglap* mRNA expressions (*P*  < 0.01, Fig. 5A). In contrast, *Enpp1*, *Spp1,* and *Slc20a1* expression was increased at the higher Pi concentrations (*P*  < 0.05, Fig. 5A). Cell viability as assessed by Alamar blue and LDH release was unaffected at all Pi concentrations tested (Supplementary Fig. 7). PHOSPHO1 and TNAP protein expressions were also inhibited by increasing Pi concentrations, whereas the addition of Pi, 3 mM and above increased the formation of mineralized bone nodules in a dose-dependent manner (*P*  < 0.001, Fig. 5B and C).

**Figure 6:**
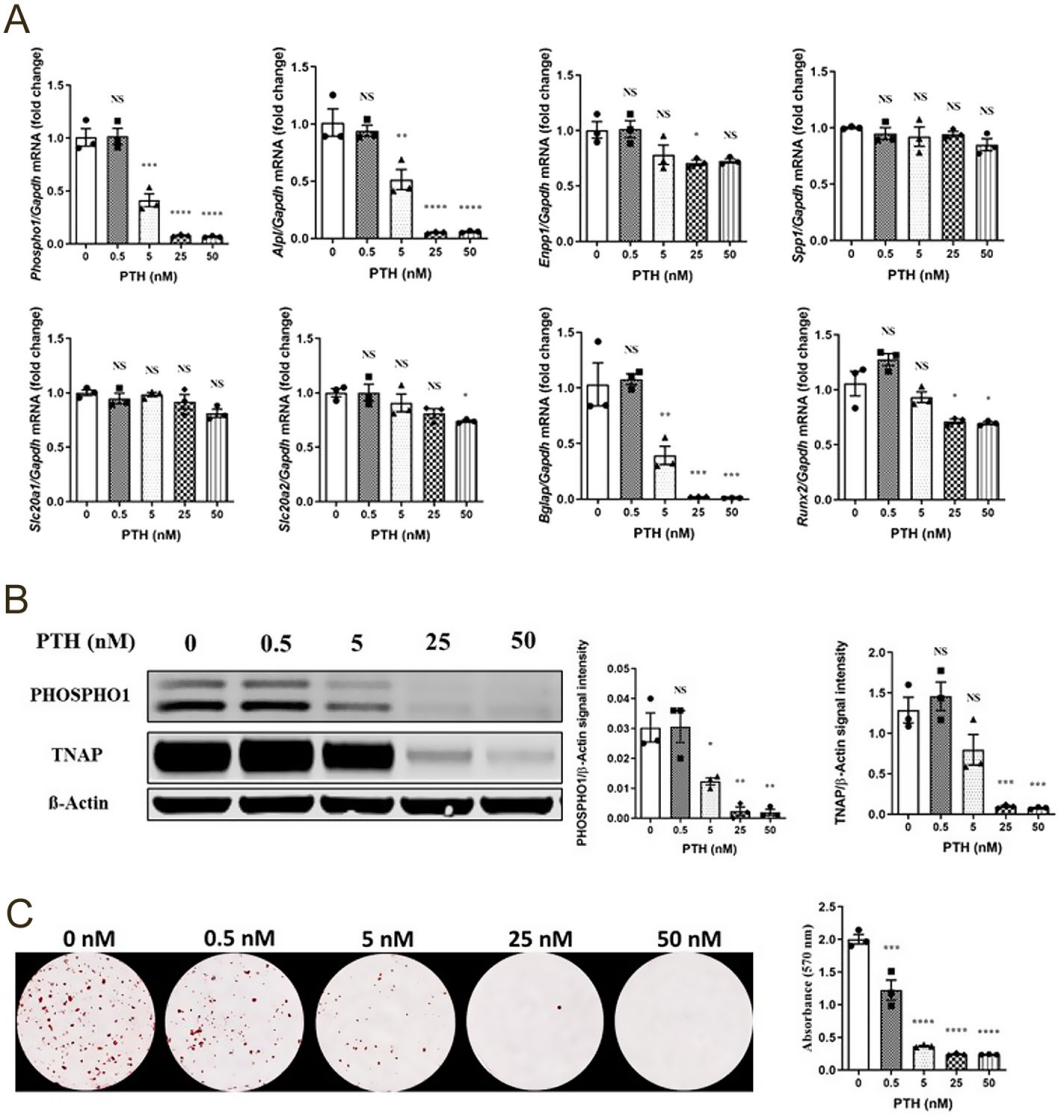
Regulation of key mineralization associated genes, proteins, and osteoblast extracellular matrix mineralization by PTH in primary osteoblasts. (A) Expression analysis of *Phospho1, Alpl, Enpp1, Spp1, Slc20a1, Slc20a2, Bglap*, and *Runx2* by osteoblasts in response to PTH (0–50 nM), (B) Western blotting analysis and quantification of PHOSPHO1 and TNAP expressions in response to PTH, and (C) representative images and quantification of Alizarin red staining in response to PTH for 28 days after confluency. PHOSPHO1 and TNAP at the gene and protein level and matrix mineralization were all decreased with increasing Pi concentrations. The data are represented as the mean ± s.e.m. (*n*  = 3); ^*^P < 0.05; ^**^P < 0.01; ^***^P < 0.001; ^****^P < 0.0001 compared to 0 nM PTH cultures.

Administration of PTH at >5 nM downregulated the expression* of Phospho1*, *Alpl,* and *Bglap* (*P*  < 0.01, Fig. 6A). *Enpp1*, *Slc20a2*, and *Runx2* expressions were also decreased but only at higher PTH concentrations (*P*  < 0.05, Fig. 6A). Reduction of PHOSPHO1 and TNAP protein expressions by increasing PTH concentrations mirrored the changes in gene expression (Fig. 6B). The addition of PTH inhibited ECM mineralization and this was noted at concentrations as low as 0.5 nM. Mineralization was completely abolished at 25 and 50 nM (*P*  < 0.001, Fig. 6C).

**Figure 7:**
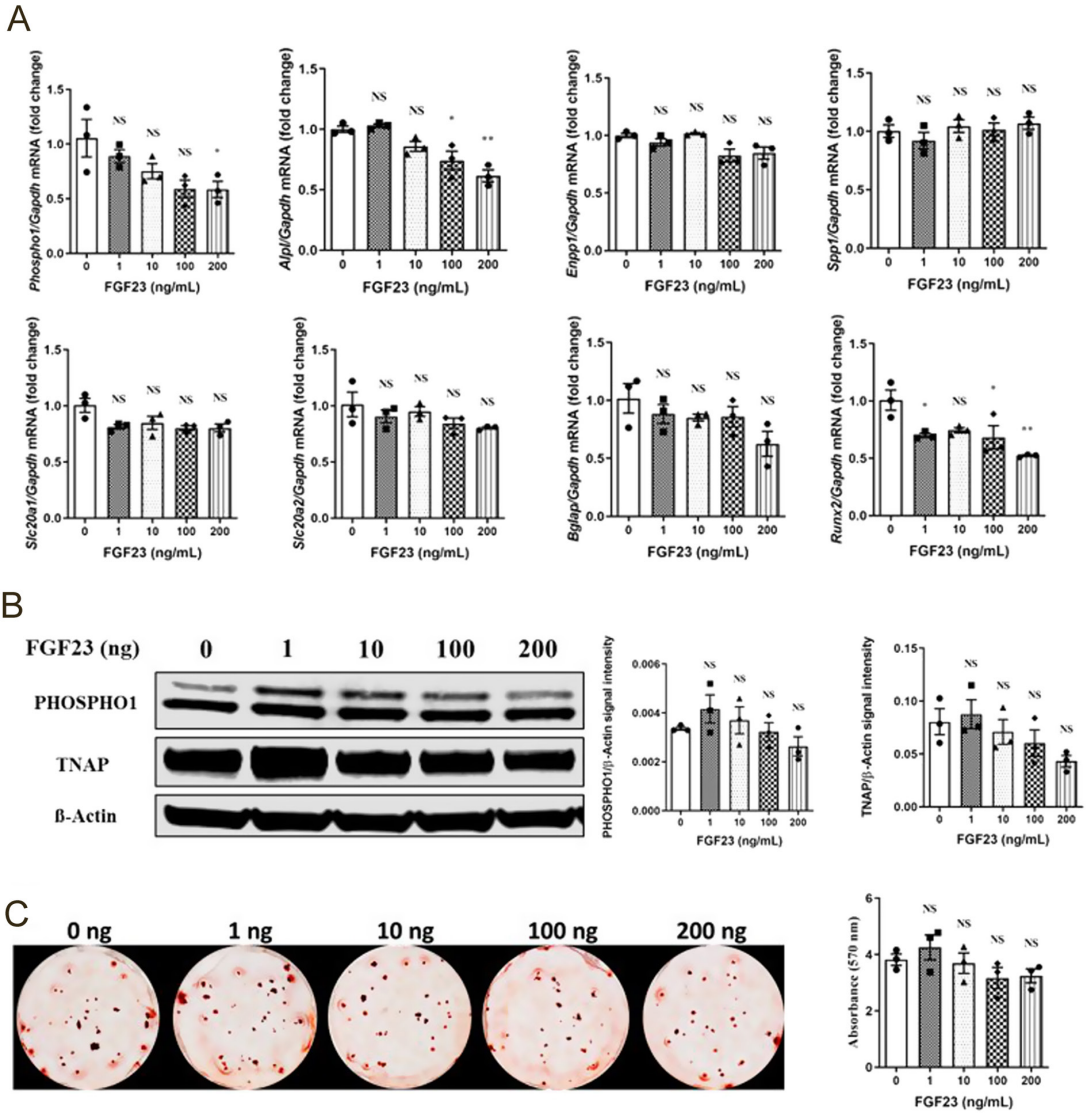
Regulation of key mineralization-associated genes, proteins, and osteoblast extracellular matrix mineralization by FGF23 in primary osteoblasts. (A) Expression analysis of *Phospho1, Alpl, Enpp1, Spp1, Slc20a1, Slc20a2, Bglap,* and *Runx2* by osteoblasts in response to FGF23 (0–200 ng/mL), (B) Western blotting analysis and quantification of PHOSPHO1 and TNAP expressions in response to FGF23, and (C) representative images and quantification of Alizarin red staining in response to FGF23 for 28 days after confluency. Phospho1 and Alpl gene expressionS were decreased at the highest FGF23 concentrations, but non-significant differences were noted with PHOSPHO1 and TNAP expression and matrix mineralization. The data are represented as the mean ± s.e.m. (*n*  = 3); ^*^P < 0.05; ^**^P < 0.01; ^***^P < 0.001; ^****^P < 0.0001 compared to 0 nM FGF23 cultures.

Exposure to FGF23 had little effect on the expression of the genes studied although both *Phospho1* and *Alpl* expressions were decreased but only at the highest FGF23 concentrations (*P*  < 0.05, Fig. 7A). The addition of Klotho to the FGF23 supplemented cultures had no further effects on gene expression when compared with FGF23 alone (data not shown). A similar trend was also noted at the protein level where PHOSPHO1 and TNAP expressions decreased in a FGF23 concentration-dependent manner, but this change did not reach statistical significance from control-treated cultures (Fig. 7B). A similar response was observed in the presence of FGF23 and Klotho (data not shown). FGF23 with or without klotho had no effects on ECM mineralization of primary osteoblasts at the concentrations tested (Fig. 7C and data not shown).

## Discussion

This study has shown that PHOSPHO1 and TNAP, two phosphatases required for bone mineralization, have altered expression in ROD. Specifically, the ROD phenotype was characterized by increased cortical BMD and this response may be mediated by increased PHOSPHO1 expression. However, the altered PHOSPHO1 expression is unlikely to be a direct result of the increased PTH, FGF23, and Pi concentrations as all decreased PHOSPHO1 expression in osteoblast cultures. The effects of uremic toxins and low calcitriol were not studied in our *in vitro* model. Nevertheless, this study is the first to implicate PHOSPHO1 function in the altered mineralization status of bones in a murine model of ROD.

In humans, deteriorating renal function contributes to the progression of ROD which results in bone loss, osteoporosis, and eventually increased morbidity and mortality resulting from fractures and/or cardiovascular disease (Gal-Moscovici & Sprague 2007). A similar bone phenotype was mirrored in this present study where cortical thinning, lower BV/TV, and increased cortical porosity were noted in the adenine-fed mice. The loss of bone is likely to be multi-factorial, but PTH-enhanced bone resorption via altered RANKL and OPG expressions is likely to predominate (Ma *et al.* 2001). In the early stages of CKD, the low bone turnover disease results from bone cell inactivity due to PTH resistance, as well as reduced calcitriol levels, and accumulation of uremic toxins (Couttenye *et al.* 1999). When renal function further deteriorates, the chronically increased PTH levels overcome peripheral PTH resistance and activate the indolent bone cells, leading to high turnover bone disease (Drüeke & Massy 2016). Bone resorption predominates in both high and low bone turnover disease and the resultant elevated serum Ca and Pi levels promote bone extra-skeletal (vascular) calcification (Zheng *et al.* 2016). In agreement with the results of this present study, others have also reported increased cortical porosity and compromised bone architecture in CKD rodent models although inconsistent effects on the cortical and trabecular compartments have been reported (Miller *et al.* 1998, Ogirima *et al.* 2006, Jia *et al.* 2013, Metzger *et al.* 2021). Although humans with CKD have been reported to have lower cortical BMD inconsistencies in trabecular and cortical BMD in CKD animal models also exist (Lau *et al.* 2013, Nickolas *et al.* 2013). Specifically, in a mouse nephrectomy model in which serum Pi levels were unchanged, trabecular and cortical BMD were increased and decreased, respectively which was the opposite of that found in this present study (Lau *et al.* 2013). The increased trabecular BMD was not influenced by dietary phosphate content, whereas the decreased cortical BMD was only noted in mice fed a high phosphate (0.9%) and not a normal phosphate (0.5%)-containing diet (Lau *et al.* 2013). In this present study, mice were fed a 0.9% phosphate-containing diet and analysis revealed that at no location along the entire cortical bone shaft was BMD lower in the CKD mice. The spectrum of bone phenotypes reported in CKD–MBD models may reflect the differing serum PTH levels at the point of study, as progressive SHPT is linked with different effects on bone quality and structure (Miller *et al.* 1998). Furthermore, whether differential expression of PHOSPHO1 and TNAP within the trabecular and cortical bone compartments contributes to the divergent BMD response is unclear and requires further investigation.

The high bone turnover status in SHPT will contribute to bone that is less mineralized, a hallmark of stage 4 and 5 CKD, and lead to reduced mechanical strength and increased risk of fractures (Drüeke & Massy 2016). Similarly, in this present study, PTH-induced skeletal remodeling is likely to, at least in part, explain the poorly mineralized trabecular bone noted in this study although PTH exposure can also inhibit osteoblast differentiation and thus indirectly delay osteoid production and matrix mineralization (Qin *et al.* 2004). Furthermore, in humans and mice, the CKD-driven increase in osteocyte secretion of Wnt/β-catenin‐signaling inhibitors such as FGF23, dickkopf 1, and sclerostin may negatively affect osteoblast function and contribute to the mineralization defect in ROD (Evenepoel *et al.* 2015, Murali *et al.* 2016*a*). The results of this present study offer changes to PHOSPHO1 and TNAP osteoblast expression as an additional/alternative explanation for the altered bone mineralization status associated with ROD.

Monitoring serum ALP has been regarded as a useful serum marker of bone turnover in ROD; however, its expression in bone, functioning as a phosphatase capable of mineralizing osteoid, has not to our knowledge been explored in the pathogenesis of ROD (Bervoets *et al.* 2003). The decreased *Alpl* expression in CKD cortical bone was not however consistent with the observed increased cortical BMD and we hypothesize that the latter is possibly driven by increased PHOSPHO1 expression which has been shown *in vitro* to promote osteoblast matrix mineralization (Huesa *et al.* 2015). To examine this further, we determined cortical BMD and other structural parameters in P1KO CKD mice. In the absence of PHOSPHO1, cortical BMD in control mice was decreased as previously reported and the increased BMD in cortical bone of CKD WT mice was not observed in the P1KO CKD mice (Yadav *et al.* 2011). Furthermore, other structural parameters such as cortical porosity, thickness, and CSA were also improved in P1KO CKD mice. It is possible that the milder cortical bone alterations noted in the CKD PHOSPHO1 KO mice are a consequence of less severe CKD phenotype in these mice. To answer this, we completed a full renal histopathological examination of the kidneys and renal scoring of tubular atrophy, protein casts, interstitial inflammation, and renal fibrosis of sections in the WT CKD mice and PHOSPHO1 KO CKD mice were similar (data not shown). Unfortunately, limited blood was obtained from the small CKD PHOSPHO1 KO mice and only serum creatinine concentrations were measured, and these did not differ between WT CKD mice (0.52 ± 0.02 mg/dL, *n*  = 4) and PHOSPHO1 KO CKD mice (0.49 ± 0.02 mg/dL, *n*  = 5) (NS). The creatinine values were also similar in the WT control (0.30 ± 0.02 mg/dL *n*  = 3) and PHOSPHO1 control (0.31 ± 0.03 mg/dL, *n*  = 8) mice. Although these data have limitations, the combined creatinine and kidney pathology data do suggest that the severity of CKD is similar in WT and PHOSPHO1 KO mice.

While supportive of our hypothesis, the mechanisms responsible for the increased PHOSPHO1 in cortical bone are unclear and cannot be explained by the direct effects of Pi, FGF23, and PTH which are all inhibitory to PHOSPHO1 expression by osteoblasts *in vitro* as shown in this study. A compensatory mechanism in an attempt to protect the bone from hypomineralization may be a possibility, but further work on this and whether PHOSPHO1 deficiency improves bone health in ROD by decreasing cortical porosity is warranted (Metzger *et al.* 2020). The decreased cortical bone TNAP expression could be a direct effect of Pi and PTH on osteoblasts as shown by the *in vitro* data of this and other *in vitro* studies (Rendenbach *et al.* 2014, Houston *et al.* 2016). Furthermore, while not observed in this study, others have reported a direct inhibitory effect of FGF23 on osteoblast matrix mineralization *in vitro* which may be mediated by decreased TNAP expression and an accumulation of PPi, via FGF receptor-3 (Shalhoub *et al.* 2011, Murali *et al.* 2016*a*). However, indirect systemic effects via disrupted vitamin D status and Pi and Ca metabolism are also likely to contribute to the altered TNAP expression in bone of CKD-MBD mice (Rendenbach *et al.* 2014, Bover *et al.* 2018).

Several studies have reported that murine mineralizing cells including cementoblasts, chondrocytes, and osteoblasts maintained in culture are sensitive to P_i_ and respond by altering the expression of mineralization-associated genes and transcription factors (Beck *et al.* 2000, Foster *et al.* 2006). The regulation of biomineralization by Pi may be related to its ability to stimulate MV release and/or the accumulation of type III NaP(i) transporter (PiT1) in osteogenic cultures promoting Pi uptake and ECM mineralization (Yoshiko *et al.* 2007, Chaudhary *et al.* 2016). Importantly, these early *in vitro* PiT1 studies used foscarnet (phosphonomethanoic acid) which has now been shown to be a non-specific inhibitor of sodium-phosphate transporters, and therefore, some caution should be taken in interpreting these results (Foster *et al.* 2006, Yoshiko *et al.* 2007, Villa-Bellosta & Sorribas 2009, Clerin *et al.* 2020). Furthermore, *in vivo* studies in mice have shown that it is not PiT-1 that is important for *in vivo* mineralization but PiT-2 (Yamada *et al.* 2018, Beck-Cormier *et al.* 2019). In relation to this present study, the availability of exogenous Pi to promote osteoblast matrix mineralization bypasses the requirement for Pi production from phosphocholine and phosphoethanolamine by PHOSPHO1 and PPi by TNAP (Houston *et al.* 2004, Roberts *et al.* 2007, Ciancaglini *et al.* 2010) and may explain the concentration-dependent decrease in PHOSPHO1 and TNAP expression by exogenous Pi which has been shown to operate in cultured cementoblasts as part of a negative feedback mechanism (Foster *et al.* 2006). In this regard, human PHOSPHO1 shares approximately 30% homology at the amino acid level with a tomato phosphate starvation-induced gene product, LePS2, which possesses phosphatase activity that can convert organic phosphorus into available Pi. Intriguingly, LePS2 expression is tightly and negatively regulated by Pi availability and is thus induced in the absence but repressed in the presence of Pi (Stenzel *et al.* 2003). It is unknown if such a Pi negative feedback mechanism controls PHOSPHO1 expression, but the increased osteoclast resorption observed in ROD will bring about the release of Pi which will contribute to the observed hyperphosphatemia and impede the skeleton from exerting its normal reservoir function when serum Pi concentrations increase (Hruska *et al.* 2008). In such a scenario, the resulting Pi stress conditions experienced by the skeleton may drive higher PHOSPHO1 expression in a similar way to the LePS2 protein and other phosphatases such as OsACP1 a PHOSPHO1-like acid phosphatase in rice (Deng *et al.* 2022).

In summary, this study has identified PHOSPHO1 as a possible mediator in the development of the cortical bone phenotype in ROD, thus providing a foundation for future research to explore potential therapies to improve bone health in CKD-MBD.

## Supplementary Material

Fig S1. Schematic view of the 5-week adenine induced CKD-MBD model and time-dependent changes in body weight. (A) Eight-week-old C57BL/6 male mice were randomly allocated to either a control (CTL; n=12) or CKD (n = 12) group. Mice in the CKD-MBD group were fed a casein-based diet containing 0.2% adenine diet for 5 weeks. Mice in the CTL group were fed a casein-based diet without adenine. (B) The bodyweight of the CKD-MBD mice progressively decreased during the 5-weeks on the adenine supplemented diet. The data are represented as the means ± SEM. **** p < 0.0001 as compared to CTL mice of the same age. Note, 4 mice in the CKD-MBD group lost more than 30% of their body weight and were removed from the study.

Fig S2. Characterization of renal pathology in CKD mouse model. (A) Representative photomicrographs of hematoxylin and eosin (H&E; i-iv), Masson’s trichrome (MT; v & vi), and von Kossa (VK; vii & viii) stained kidney sections from CTL and CKD mice at end of the study (13 weeks of age). (i & ii) kidney sections showing gross pathology, (d) atrophic tubuli with protein casts (green arrows) and dilated Bowman’s space (blue arrow). (f) Dilated tubules (green arrows) and interstitial fibrosis (blue arrows. (h) Calcification of tubular structures (blue arrows). Scale bar, (i & ii) 500 μm, (iii-viii) 100 μm. (B) Renal scoring of tubular atrophy, protein casts, interstitial inflammation, and renal fibrosis of sections from 4 CTL and 4 CKD mice. All indices were higher in kidneys from CKD-MBD mice. Renal scoring scale: 0 = normal; 1 = mild, involvement of <25% of the cortex; 2 = moderate, involvement of 25 to 50% of the cortex; 3 = severe, involvement of 50 to 75% of the cortex; 4 = extensive damage involving >75% of the cortex. The data are represented as the mean ± SEM (n = 4); **** p < 0.0001.

Fig S3. Expression levels of injury associated markers in kidneys of CTL and CKD mice. (A) Fgf23, Spp1 and Lcn2 expression was higher whereas Slc34a1 was lower in kidneys of CKD-MBD mice at end of the study (13 weeks of age). Four random samples from each of the CTL and CKD groups were selected for analysis. (B) Representative western blot of osteopontin (OPN) and type II sodium-phosphate cotransporter (NaPi-2a) protein expression from BBMV of kidneys at end of study (13 weeks of age). The increased osteopontin and decreased NaPi-2a in CKD-MBD kidneys confirm gene expression data. The data are represented as the mean ± SEM (n = 4); * p < 0.05; ** p < 0.01; *** p < 0.001; **** p < 0.0001

Fig S4. Histological characterization of trabecular bone in CKD mice (A) Representative photomicrographs of tibia sections stained for hematoxylin and eosin (H&E; i & ii) and Goldner’s trichrome (iii & iv) and reacted for tartrate acid phosphatase activity (TRAP; v & vi; blue arrow) from CTL and CKD-MBD mice at end of study (13 weeks of age). Scale bar, 100 μm. (B) Bone volume/tissue volume (BV/TV) was decreased in CKB-MBD mice whereas osteoid volume/bone volume (OV/BV); osteoclast surface/bone surface (Oc.S/BS); number of osteoclasts/bone surface (N.Oc/BS) were all increased in CKD-MBD mice. The data are represented as the mean ± SEM (n = 6); * p < 0.05; ** p < 0.01.

Fig S5. Micro-CT analysis of trabecular bone of wild-type (WT) and PHOSPHO1 deficient CTL and CKD mice. Tb. BMD, Tb. BV/TV, Tb. N and Tb. Conn. Tb. were increased in PHOSPHO1 deficient CKD-MBD tibia when compared to their respective WT CKD-MBD tibia. The data are represented as the mean ± SEM (n = 8); * p < 0.05; ** p < 0.01; ** p < 0.001; **** p < 0.0001 compared to WT CTL bones.

Fig S6. Characterization of osteoblast culture model showing temporal increases in extracellular matrix mineralization and PHOSPHO1 and TNAP expression. (A) Alizarin red staining, (B) quantification of matrix mineralization (C) RT-qPCR analysis of Phospho1, and Alpl mRNA expression, (D) western blot analysis, and (E) quantification of PHOSPHO1, and TNAP expression and by primary osteoblasts cultured in the basal Pi-substrate free mineralization medium over a 28-day culture period. The data are represented as the mean ± SEM (n = 3); * p < 0.05; ** p < 0.01; *** p < 0.001; **** p < 0.0001 in comparison with day 0, post confluence cultures.

Fig S7. The effect of Pi on osteoblast viability. Cells were exposed to Pi (1-5 mM) for 28 days after confluency and viability were assessed by (A) Alamar Blue assay, and (B) LDH assay. Cell viability was not affected by Pi at all concentrations tested. The data are represented as the mean ± SEM (n ≥ 3); NS, not significance from the 1 mM control group.

Table S1. Sequences of primers used for qPCR

Table S2. Primary antibodies used for western blotting

Table S3. Secondary antibodies used for western blotting

## Declaration of interest

The authors declare that there is no conflict of interest that could be perceived as prejudicing the impartiality of the research reported.

## Funding

This work was funded by the Tri-Service General Hospital http://dx.doi.org/10.13039/501100010425 (TSGH), National Defense Medical Center (NDMC), Taiwan, through a Ph.D. scholarship award to S-N H. The authors are also grateful to the Biotechnology and Biological Sciences Research Council http://dx.doi.org/10.13039/501100000268 (BBSRC) for Institute Strategic Programme Grant Funding BB/J004316/1 to C F, L A S and V E M, grant DE12889 from the National Institute of Dental and Craniofacial Research http://dx.doi.org/10.13039/100000072 (NIDCR) to J L M and Versus Arthritis http://dx.doi.org/10.13039/501100012041 (grant 20581) awarded to A A P to support B J. For the purpose of open access, the author has applied a CC BY public copyright license to any Author Accepted Manuscript version arising from this submission.

## Ethical approval

All experimental protocols were approved by Roslin Institute’s Animal Users Committee and the animals were maintained in accordance with UK Home Office guidelines for the care and use of laboratory animals, and with the ARRIVE guidelines.

## Author contribution statement

Shun-Neng Hsu: Conceptualization, Formal Analysis, Methodology, Investigation, Writing – Original draft, Funding acquisition. Louise A Stephen: Formal Analysis, Methodology, Investigation, Supervision, Writing – Review and Editing. Scott Dillon: Formal Analysis, Methodology, Investigation. Elspeth Milne: Formal Analysis, Methodology. Behzad Javaheri: Formal Analysis, Methodology. Andrew A Pitsillides: Methodology, Investigation. Amanda Novak: Conceptualization, Methodology. Jose Luis Millán: Investigation. Vicky E Macrae: Conceptualization, Supervision, Funding acquisition; Writing – Review and Editing. Katherine A Staines: Conceptualization, Investigation, Supervision, Writing – Review and Editing, Funding acquisition. Colin Farquharson: Conceptualization, Investigation, Writing – Review and Editing, Supervision, Funding acquisition. All authors approved the final version of the manuscript.
