# Supplementary figures and images for: Increased PHOSPHO1 expression mediates cortical bone mineral density in renal osteodystrophy
Source: J Endocrinol. 2022 Jul 25;254(3):167–81. doi: 10.1530/JOE-22-0097 (PMC9422252; doi:10.1530/JOE-22-0097)

**A**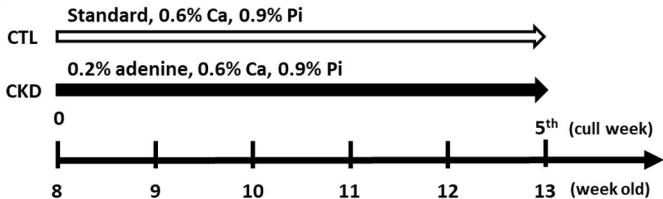**B**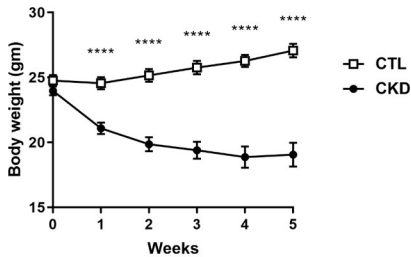

Supplement: Fig S1. Schematic view of the 5-week adenine induced CKD-MBD model and time-dependent changes in body weight. (A) Eight-week-old C57BL/6 male mice were randomly allocated to either a control (CTL; n=12) or CKD (n = 12) group. Mice in the CKD-MBD group were fed a casein-based diet containing 0.2% ade [file supplementary_figure_1.pdf]

A

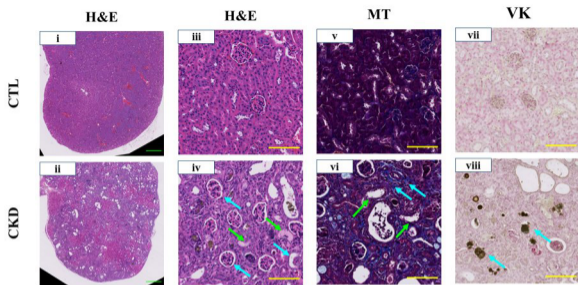

B

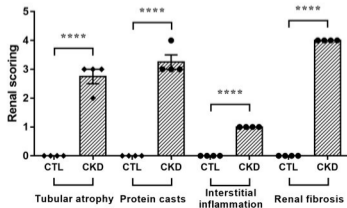

Supplement: Fig S2. Characterization of renal pathology in CKD mouse model. (A) Representative photomicrographs of hematoxylin and eosin (H&E; i-iv), Masson’s trichrome (MT; v & vi), and von Kossa (VK; vii & viii) stained kidney sections from CTL and CKD mice at end of the study (13 weeks of age). (i & ii) kidn [file supplementary_figure_2.pdf]

**A**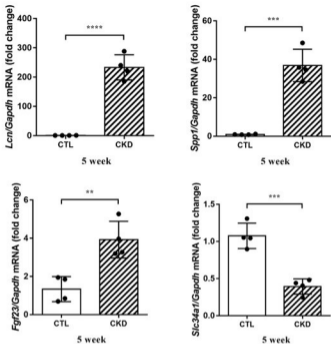**B**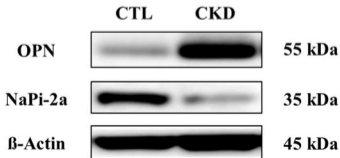

Supplement: Fig S3. Expression levels of injury associated markers in kidneys of CTL and CKD mice. (A) Fgf23, Spp1 and Lcn2 expression was higher whereas Slc34a1 was lower in kidneys of CKD-MBD mice at end of the study (13 weeks of age). Four random samples from each of the CTL and CKD groups were selected for  [file supplementary_figure_3.pdf]

**A****H&E****Goldner's Trichrome****TRAP****CTL**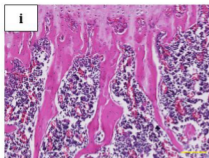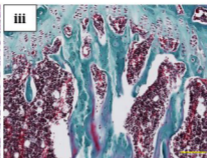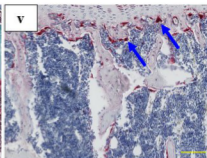**CKD**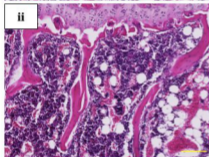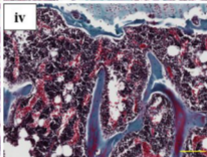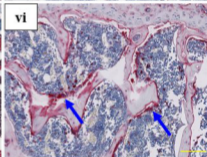**B**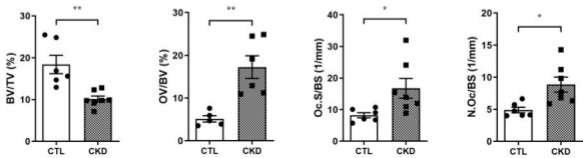

Supplement: Fig S4. Histological characterization of trabecular bone in CKD mice (A) Representative photomicrographs of tibia sections stained for hematoxylin and eosin (H&E; i & ii) and Goldner’s trichrome (iii & iv) and reacted for tartrate acid phosphatase activity (TRAP; v & vi; blue arrow) from CTL and CKD [file supplementary_figure_4.pdf]

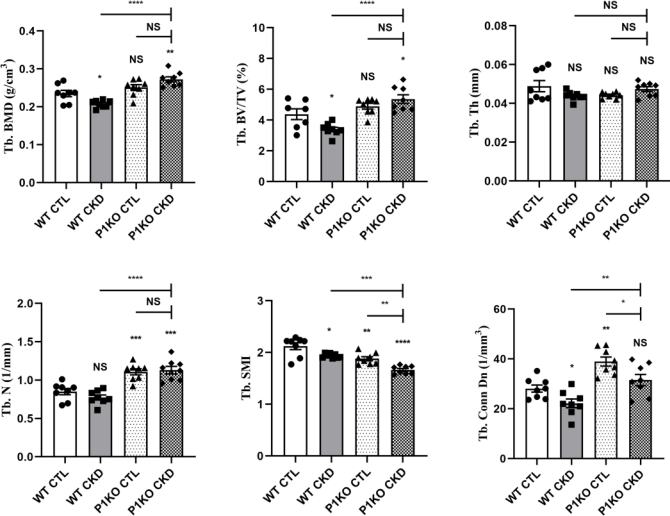

Supplement: Fig S5. Micro-CT analysis of trabecular bone of wild-type (WT) and PHOSPHO1 deficient CTL and CKD mice. Tb. BMD, Tb. BV/TV, Tb. N and Tb. Conn. Tb. were increased in PHOSPHO1 deficient CKD-MBD tibia when compared to their respective WT CKD-MBD tibia. The data are represented as the mean ± SEM (n = 8 [file supplementary_figure_5.pdf]

**A**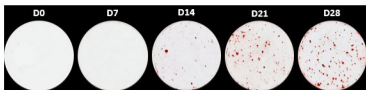**B**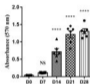**C**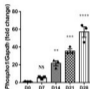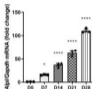**D**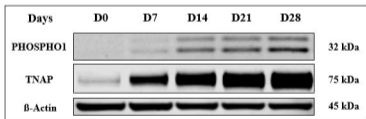**E**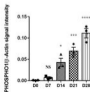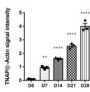

Supplement: Fig S6. Characterization of osteoblast culture model showing temporal increases in extracellular matrix mineralization and PHOSPHO1 and TNAP expression. (A) Alizarin red staining, (B) quantification of matrix mineralization (C) RT-qPCR analysis of Phospho1, and Alpl mRNA expression, (D) western blot [file supplementary_figure_6.pdf]

**A**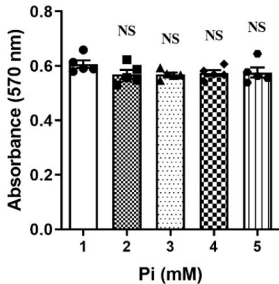**B**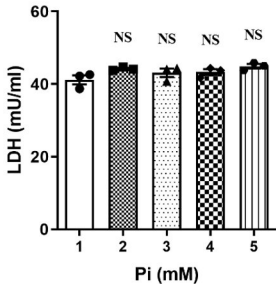

Supplement: Fig S7. The effect of Pi on osteoblast viability. Cells were exposed to Pi (1-5 mM) for 28 days after confluency and viability were assessed by (A) Alamar Blue assay, and (B) LDH assay. Cell viability was not affected by Pi at all concentrations tested. The data are represented as the mean ± SEM (n  [file supplementary_figure_7.pdf]
