# Supplementary material for: Increased PHOSPHO1 expression mediates cortical bone mineral density in renal osteodystrophy
Source: J Endocrinol. 2022 Jul 25;254(3):167–81. doi: 10.1530/JOE-22-0097 (PMC9422252; doi:10.1530/JOE-22-0097)
Supplement: Table S1. Sequences of primers used for qPCR [file supplementary_table_1.pdf]

Table S1. Sequences of primers used for qPCR

Genes	Forward, 5'→ 3'	Reverse, 5'→ 3'	Source
Atp5b	ATGCCACTTCCAAGGTAGCG	GCAACAGTCAGACCAGTCAGA	Primer Design
Gapdh	AAATGGTGAAGGTCGGTGTG	TGAAGGGGTCGTTGATGG	Sigma-Aldrich
Phospho1	TTCTCATTTCGGATGCCAACA	TGAGGATGCGGCGGAATAA	Eurofins Genomics
Alpl	GGGACGAATCTCAGGGTACA	AGTAACTGGGGTCTCTCTC	Sigma-Aldrich
Enpp1	GCTAATCATCAGGAGGTCAAG	CTGGTAGAATCCCGTCAATC	Sigma-Aldrich
Spp1	CACTCCAATCGTCCCTACAGT	CTGGAAACTCCTAGACTTTGACC	Sigma-Aldrich
Slc20a1	TGTGGCAAATGGGCAGAAG	AGAAAGCAGCGGAGAGACGA	Sigma-Aldrich
Slc20a2	CCATCGGCTTCTCACTCGT	AAACCAGGAGGCGACAATCT	Sigma-Aldrich
Anxa6	GGACCTCATCGAAGACTTGAAG	CTTTGGCGTCACAATAGGCAA	Sigma-Aldrich
Ank	TCGCTGCCTTCCCTTTTATG	GGTGACTGTGAAGCAAAATGG	Sigma-Aldrich
Npnt	TGCCCTATCGTGTTCCATG	ACTCTTCCAGTCGCACATTC	Sigma-Aldrich
Pdpn	AACAAGTCACCCCAATAGAGATAAT	CTAACAAGACGCCAACTATGATTC	Sigma-Aldrich
Fgf23	GGATCTCCACGGCAACATTT	GTAGTGATGCTTCTGCGACAA	Eurofins Genomics
Bglap	CCGGGAGCAGTGTGAGCTTA	TAGATGCGTTTGTAGGCGGTC	Sigma-Aldrich
Runx2	ACCATAACAGTCTTCACAAATCCT	CAGGCGATCAGAGAACAAACTA	Sigma-Aldrich
Sost	TGAGAACAACCAGACCATGAAC	TCAGGAAGCGGGTGTAGTG	Primer Design
Bmp2	TCAAGCCAAACACAAACAGC	AGCCACAATCCAGTCATTCC	Sigma-Aldrich
Klotho	GGACAATGGCTTTCCTCCTT	TGCACATCCCACAGATAGACA	Sigma-Aldrich
Adipoq	AAGAAGGACAAGGCCGTTCTCTT	GCTATGGGTAGTTGCAGTCAGTT	Sigma-Aldrich
