# Supplementary material for: Increased PHOSPHO1 expression mediates cortical bone mineral density in renal osteodystrophy
Source: J Endocrinol. 2022 Jul 25;254(3):167–81. doi: 10.1530/JOE-22-0097 (PMC9422252; doi:10.1530/JOE-22-0097)
Supplement: Table S2. Primary antibodies used for western blotting [file supplementary_table_2.pdf]

Table S2. Primary antibodies used for western blotting

Target	Source	Company	Dilution	Molecular weight
β-actin	Rabbit	Cell Signaling Technology	1:4000	45 kDa
PHOSPHO1	Human	AbD Serotec and Bio-Rad	1:500	32 kDa
TNAP	Rat	R&D Systems	1:1000	75 kDa
