# Supplementary material for: Increased PHOSPHO1 expression mediates cortical bone mineral density in renal osteodystrophy
Source: J Endocrinol. 2022 Jul 25;254(3):167–81. doi: 10.1530/JOE-22-0097 (PMC9422252; doi:10.1530/JOE-22-0097)
Supplement: Table S3. Secondary antibodies used for western blotting [file supplementary_table_3.pdf]

**Table S3. Secondary antibodies used for western blotting**

| Target | Source | Company | Dilution |
|---|---|---|---|
| β-actin | Goat anti-rabbit | Dako | 1:1000 |
| PHOSPHO1 | Goat anti-human | Bio-Rad | 1:1000 |
| TNAP | Goat anti-rat | R&D Systems | 1:1000 |
| β-actin | Donkey anti-rabbit | LI-COR Biosciences | 1:15000 |
| PHOSPHO1 | Goat anti-human | LI-COR Biosciences | 1:15000 |
| TNAP | Goat anti-rat | LI-COR Biosciences | 1:15000 |
